# Height system connection between island and mainland using a hydrodynamic model: a case study connecting the Dutch Wadden islands to the Amsterdam ordnance datum (NAP)

**DOI:** 10.1007/s00190-018-1133-3

**Published:** 2018-03-09

**Authors:** D. C. Slobbe, R. Klees, M. Verlaan, F. Zijl, B. Alberts, H. H. Farahani

**Affiliations:** 1 Delft University of Technology, Stevinweg 1, 2628 CN Delft, The Netherlands; 2 Deltares, Boussinesqweg 1, 2629 HV Delft, The Netherlands; 3 Rijkswaterstaat Centrale Informatievoorziening, Derde Werelddreef 1, 2622 HA Delft, The Netherlands

**Keywords:** Hydrodynamic leveling, Height system, Tide gauge, Mean water level

## Abstract

We present an efficient and flexible alternative method to connect islands and offshore tide gauges with the height system on land. The method uses a regional, high-resolution hydrodynamic model that provides total water levels. From the model, we obtain the differences in mean water level (MWL) between tide gauges at the mainland and at the islands or offshore platforms. Adding them to the MWL relative to the national height system at the mainland’s tide gauges realizes a connection of the island and offshore platforms with the height system on the mainland. Numerical results are presented for the connection of the Dutch Wadden islands with the national height system (Normaal Amsterdams Peil, NAP). Several choices of the period over which the MWLs are computed are tested and validated. The best results were obtained when we computed the MWL only over the summer months of our 19-year simulation period. Based on this strategy, the percentage of connections for which the absolute differences between the observation- and model-derived MWL differences are $\le 1$ cm is about 34% (46 out of 135 possible leveling connections). In this case, for each Wadden island we can find several connections that allow the transfer of NAP with (sub-)centimeter accuracy.

## Introduction

Traditionally, nearby islands are connected to the mainland height system using hydrostatic leveling (e.g., Waalewijn 1964; Sneddon 1972). This highly specialized measurement technique, which has been applied to cross water bodies up to distances of about 20 km (Andersen 1992), is very expensive and time-consuming. This is the reason why most countries in the world decided to abandon this technique. In the Netherlands, this happened in 2002. This decision was prompted by the expectation that in near future GNSS/leveling (e.g., Schwarz et al. 1987) could be used for height system connection. Contrary to hydrostatic leveling, GNSS/leveling can be applied over arbitrary distances provided the (quasi-)geoid model has sufficient coverage. Unfortunately, however, the application of GNSS/leveling *still* suffers from errors in the regional quasi-geoid models which even in northwest Europe easily attain values of several centimeters or more (Farahani et al. 2017a). Part of these errors are long-wavelength and maybe reduced using the data to be acquired during the upcoming GRACE follow-on mission. At the same time, Farahani et al. (2017b) showed that computing a sub-centimeter quasi-geoid model for the Netherlands mainland and continental shelf in terms of omission and commission errors requires better high-resolution data in the marine areas than currently available. They concluded that the only available and at the same time cost-effective acquisition technique that is able to provide the gravity data at sea with sufficient density and spatial resolution is airborne gravimetry. Getting the funds for an airborne gravity campaign is, however, not realistic in the short term. As such, an *operational* method is lacking that allows the maintenance of height systems at the centimeter level. The practical consequence for the Netherlands is that since 2002 the Wadden islands and offshore stations have not been connected to the NAP height system.

Bonnefond et al. (2003) proposed the use of pelagic GNSS surveys to determine the local marine geoid slope between offshore altimetric measurements and coastal tide gauge locations. Here, GNSS is used to determine the ellipsoidal heights of the instantaneous sea surface. After subtracting the water levels observed at a nearby tide gauge, geoid heights are obtained from which the slope can be determined. To be a candidate technique for the problem at hand, this nearby tide gauge should be connected to the mainland height system. The complex hydrodynamics inside the Wadden Sea, however, do not allow to extrapolate these corrections up to the Wadden islands. Not to mention that Bonnefond et al. (2003) did not achieve the centimeter accuracy level we need. Therefore, we do not consider this technique as a candidate technique to connect the Wadden islands to the NAP height system.

The only alternative realizable in the short term is to use “hydrodynamic leveling” or “ocean leveling” (e.g., Proudman 1953; Cartwright and Crease 1963; Woodworth et al. 2013). It is applied between tide gauges and requires knowledge of the difference in the mean dynamic topography (MDT) between them. Different variants in deriving such differences have been published. Cartwright and Crease (1963) used a 1D hydrodynamic model fed by local measurements of the atmospheric pressure, wind speeds, and electrical potential across a submarine cable. Over a distance of 70 km, they obtained an accuracy of 1.5 cm. Wübbelmann (1992) achieved a comparable accuracy for a 20 km connection over the Fehmarn Belt. Woodworth et al. (2013) compared along the European and American coastlines of the North Atlantic and the North American Pacific coast and in the Mediterranean, the use of observation-derived MDTs (both GNSS/leveling and altimeter) to estimates obtained with global ocean circulation models and reported consistency at the sub-decimeter level. Finally, Liibusk et al. (2013) compared the use of an altimeter-derived MDT model to MDT differences derived with GNSS/leveling in a small and semi-enclosed water body. They reported discrepancies that exceed the range of errors of the pressure gauges they used. Therefore, they concluded that in a small and semi-enclosed water body it could be safer to ignore the MDT differences.

These variants are all suboptimal. The method used by Cartwright and Crease (1963) requires special infrastructure, which makes it too costly in case this infrastructure is not available. The use of a satellite radar altimeter-derived MDT model in coastal areas is not appropriate due to a lack of reliable altimetry data (e.g., Vignudelli et al. 2011) and the lack of an operational solution to make the mean sea surface spectrally consistent with the geoid (e.g., Slobbe et al. 2012). Finally, global circulation models are not suitable to derive the MDT in shelf seas and coastal waters. As already pointed out by Woodworth et al. (2013), these models are designed to study the deep ocean circulation rather than sea level changes at the coast. In shelf seas and coastal waters, they lack the required spatial and temporal resolutions as well as relevant forcing factors.

Here, we propose to use a regional, high-resolution hydrodynamic model that provides total water levels to compute mean water level (MWL) differences between tide gauge stations. The term MWL is used in favor of the term MDT to express the freedom we have in defining the averaging period; strictly speaking, to conduct the leveling modeled water levels are only needed at one epoch (i.e., no averaging is needed at all). The use of a regional, high-resolution hydrodynamic model will solve the deficiencies regarding the spatial and temporal resolutions and forcing factors inherent to the usage of an ocean circulation model as identified by Woodworth et al. (2013). Compared to Cartwright and Crease (1963) the advantages are twofold. First, we obtain the MWL differences among all *N* tide gauges located within the model domain in one model run (from these we can select $N-1$ independent connections). Second, no electrical potential measurements are required to estimate the mean longitudinal currents. These advantages make the method, which we will refer to as “*model-based hydrodynamic leveling*”, very flexible and efficient. In case a regional, high-resolution hydrodynamic model is already available, the method can also be considered as cost-effective. In many coastal countries, we expect this to be the case, as such models are a key element in coastal flood forecasting systems.

A rigorous implementation of this method requires access to a hydrodynamic model that resolves all *3D* physical processes that contribute to the MWL. Unfortunately, the current *operational* hydrodynamic models in the Netherlands are barotropic (a baroclinic model is under development). These 2D models are primarily developed to forecast water level (tide and surge) *variations* under day-to-day conditions as well as during storm surges. As the water level variations in coastal regions due to time-varying baroclinic pressure gradients (e.g., due to freshwater discharge or salinity intrusion in rivers and estuaries) are much smaller than the dominating tide and surge ones, these are justifiably neglected when forecasting storm surges. Since we are interested in the MWL, however, neglecting the baroclinic processes is no longer valid. Even though we lack access to a 3D model, we can include a first order estimate of the baroclinic signal using the approach presented and validated by Slobbe et al. (2013a). Indeed, a proper representation of strong local baroclinic effects can only be achieved with a 3D model. Remember, however, that the method provides more leveling connections than strictly needed to transfer the mainland height system to islands/offshore platforms. Therefore, we do not need to obtain a good representation at *all* tide gauge locations; those where the MWL is dominated by local baroclinic signals will be excluded.

The main research question addressed in this study is whether hydrodynamic leveling based on a regional, high-resolution 2D hydrodynamic model extended to account for depth-averaged water density variations allows to transfer NAP from the Dutch mainland to the Wadden islands and nearby offshore platforms *with centimeter accuracy*. The answer to this question is also of interest to other ocean areas, because it shows what can be achieved with a computationally efficient model. Section 2 presents the model used in this study. In Sect. 3, we discuss how the tide gauges are selected between which the differences in MWL are computed. This selection is, among others, required to address the limitations of our model in resolving the local MWL at some locations. Thereafter, we present and discuss the results of the numerical experiments (Sect. 4). Finally, we conclude by emphasizing the main findings and identifying topics for future research.

## The hydrodynamic models and forcing data

In this study, we used a model that consists of two domains that are coupled by means of domain decomposition. In the outer domain, the Dutch Continental Shelf Model version 6 (DCSMv6) (Zijl et al. 2013) is used. In the inner domain, the Zuidelijk Noordzee model version 4 (ZUNOv4) (Zijl et al. 2015). The model, referred to as “DCSMv6-ZUNOv4”, is developed as an application of the WAQUA software package which solves the depth-integrated shallow-water equations for hydrodynamic modeling of free-surface flows (Leendertse 1967; Stelling 1984). In this section, we briefly introduce the model and the forcing data used to obtain the model-derived MWL.

### Computational grid

**Fig. 1:**
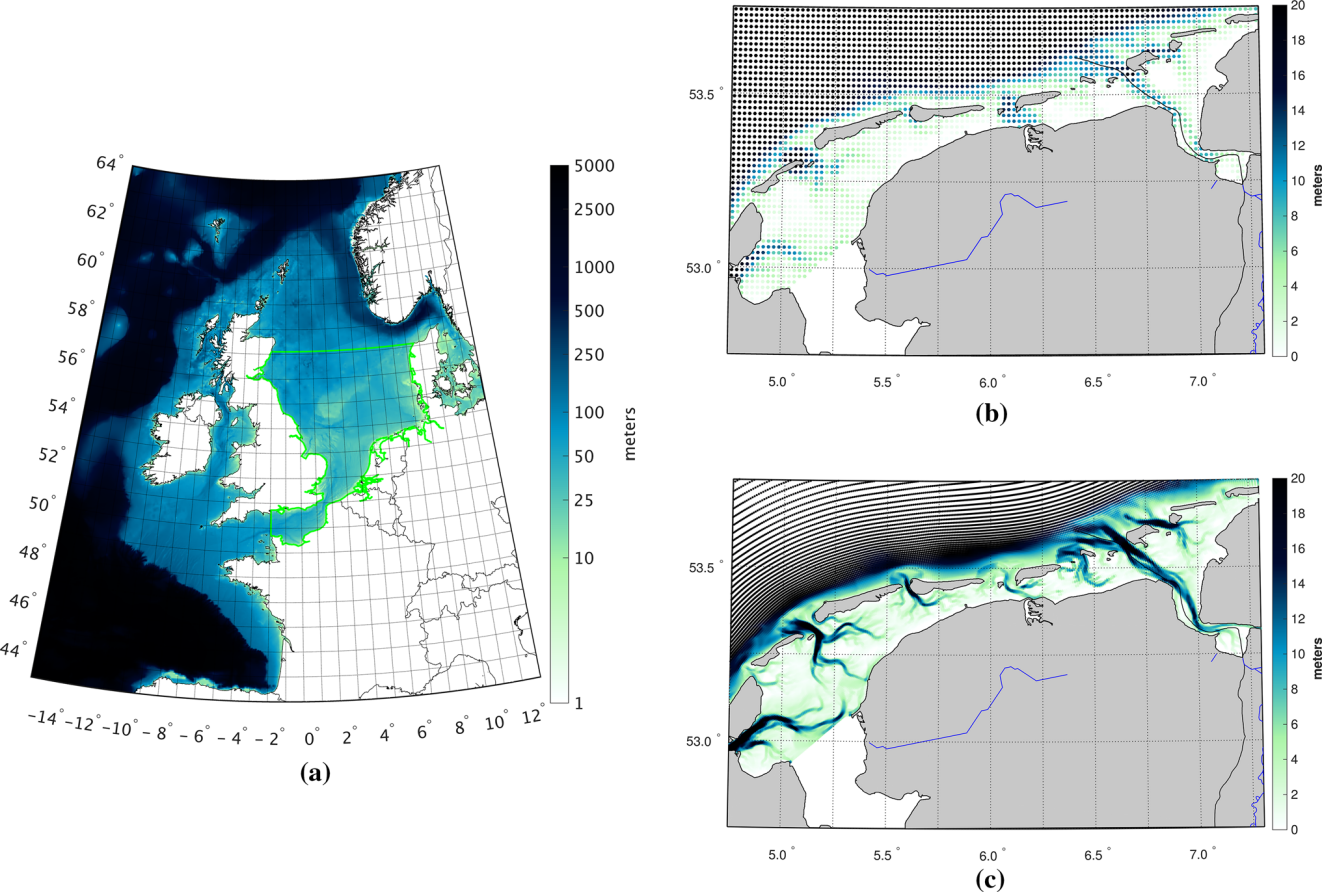
The DCSMv6-ZUNOv4 model domain and bathymetry (**a**). The green line outlines the domain comprised by the ZUNOv4 model. The right panels show the schematization of the Wadden Sea by the DCSMv6 (**b**) and the ZUNOv4 model (**c**). Node colors represent the bathymetry

The DCSMv6 is the current operational 2D storm surge model that is recently developed as part of a comprehensive study to improve water level forecasting in Dutch coastal waters. The model covers the part of the northwest European continental shelf between $15^\circ$W to $13^\circ$E and $43^\circ$N to $64^\circ$N. It has a uniform horizontal resolution of $1/40^\circ$ in east–west direction and $1/60^\circ$ in north–south direction, which corresponds to a grid cell size of about $1 \times 1$ nautical miles. Figure 1a shows the model domain and bathymetry. The ZUNOv4 comprises the southern North Sea and eastern English Channel (see Fig. 1a for an outline of the model domain). Contrary to the DCSMv6, it has a variable grid size ranging from about 2000 m down to 200–400 m in the Dutch estuaries and the Wadden Sea (cf. Fig. 1b, c).

### Forcing

In our experiments, three forcing terms are considered: the tide-generating forcing, the forcing induced by wind and mean sea level pressure variations, and the forcing induced by the depth-averaged horizontal variations in water density (referred to as the “baroclinic forcing”). Like most models, the DCSMv6-ZUNOv4 uses the Boussinesq approximation and therefore conserves volume and not mass. This implies that it cannot reproduce the net expansion/ contraction of the oceans due to heating/ cooling (Greatbatch 1994). There are “pragmatic” solutions to include this signal (Mellor and Ezer 1995; Slobbe et al. 2013a) via the water levels prescribed at the open sea boundaries. However, for the application we have in mind the contribution can be ignored as the correction cancels out when computing the MWL *differences* between tide gauges.

#### Tide-generating forcing

The tide-generating forces account for the components of the tide with a Doodson number ranging from 55.565 to 375.575. Given the size of the model domain, these cannot be neglected; the forces induce water level variations up to 10 cm (Zijl et al. 2013).

#### Atmospheric wind and mean sea level pressure

Atmospheric wind and mean sea level pressure data are obtained from the publicly available data of the interim reanalysis project ERA-Interim (Dee et al. 2011) provided by the European Centre for Medium-Range Weather Forecasts. ERA-Interim covers the period from January 1, 1979 onwards and provides three-hourly grids with a spatial resolution of $0.75^\circ \times 0.75^\circ$.

The wind and air pressure fields are interpolated to the DCSMv6-ZUNOv4 grid using the Generic Mapping Tools (GMT) greenspline routine (Wessel 2009) with a tension factor of 0. To account for the differences between on- and offshore wind regimes, we only used the grid cells that are flagged as “sea” in the ERA-Interim land/sea mask. To convert the wind speeds to wind stresses, we used the Charnock drag formulation (Charnock 1955). This formulation includes the so-called Charnock coefficient; a dimensionless bulk parameter depending upon atmospheric conditions and surface wave parameters that determines the grip of the wind on the sea surface. In our simulations, we used the time/space-varying Charnock coefficients that are part of the ERA-Interim output.

#### Baroclinic forcing

In operational use, the DCSMv6-ZUNOv4 includes tides and meteorological forcing only; baroclinic processes are ignored. This is a valid simplification for storm surge predictions a few days ahead as the short-term water level variations induced by baroclinic effects are negligible compared to those induced by tides, winds, and atmospheric pressure variations (especially on the continental shelf). In this study, however, we aim to reconstruct MWLs over a period up to 19 years, which does comprise a significant baroclinic contribution (Slobbe et al. 2013a). Therefore, we extended the DCSMv6-ZUNOv4 to account for depth-averaged horizontal variations in water density using the method described by Slobbe et al. (2013a). A key feature of that methodology is that the depth-averaged horizontal baroclinic pressure gradients are treated as a *diagnostic variable* in the model simulations.

The pressure gradient fields are computed from temperature and salinity fields obtained from a reanalysis with a 3D hydrodynamic model defined on a larger domain than the DCSMv6. In this study, we used the 3D daily mean temperature and salinity fields from the Atlantic—European North West Shelf—Ocean Physics Reanalysis conducted by the UK Met Office (Wakelin et al. 2016), available at the Copernicus Marine Environment Monitoring Service (http://marine.copernicus.eu/). This reanalysis covers the period January 1985–July 2014 and is based upon the Forecasting Ocean Assimilation Model 7 km Atlantic Margin Model; a hydrodynamic model of the North West European shelf forced at the surface by ERA-interim winds, atmospheric temperature, and precipitation fluxes. The overall procedure to compute the depth-averaged horizontal baroclinic pressure gradients is a slightly modified version of Slobbe et al. (2013a) and consists of 4 steps.

**Step 1**. *Transform salinity and temperature to water density.* The daily mean salinity and temperature fields exploited in this study are available at 24 geopotential (z-level) vertical levels based upon the standard depths of the International Council for the Exploration of the Sea. To transform them to water density fields, we used the international thermodynamic equation of seawater 2010 (IOC, SCOR and IAPSO 2010);

**Step 2**. *Upsample the density profiles.* The separation between the vertical levels increases with increasing depth. Using a spline interpolation, we upsampled each density profile such that the maximum sampling interval is 25 m;

**Step 3**. *Compute the depth-averaged horizontal baroclinic pressure gradients.* The pressure gradients in *x* (east–west) and *y* (north–south) directions, $\Gamma _{x}$ and $\Gamma _{y}$, respectively, are computed using (Slobbe et al. 2013a, Eq. 5):

1 $$\begin{aligned} \Gamma _{x}&= \sum \limits _{l=1}^{L}\frac{h_l}{d}\left( \frac{g}{\rho _l}\left\{ \frac{h_l}{2}\frac{\partial \rho _l}{\partial x} +\sum _{j=1}^{l-1}\left( h_j\frac{\partial \rho _j}{\partial x}+(\rho _j-\rho _l)\frac{\partial h_j}{\partial x}\right) \right\} \right) ,\nonumber \\ \Gamma _{y}&= \sum \limits _{l=1}^{L}\frac{h_l}{d}\left( \frac{g}{\rho _l}\left\{ \frac{h_l}{2}\frac{\partial \rho _l}{\partial y} +\sum _{j=1}^{l-1}\left( h_j\frac{\partial \rho _j}{\partial y}+(\rho _j-\rho _l)\frac{\partial h_j}{\partial y}\right) \right\} \right) . \end{aligned}$$

where *d* is the depth below the reference surface, *g* the gravity acceleration (assumed to be constant), *L* the number of depth layers in the 3D model from which the temperature and salinity fields are derived, *j* and *l* the layer indices, $h_j$ and $h_l$ the thickness of layers *j* and *l*, and $\rho _j$ and $\rho _l$ the mean water density of layers *j* and *l*.

**Step 4**. *Interpolate the depth-averaged horizontal baroclinic pressure gradients to the DCSMv6-ZUNOv4 grid nodes.* The interpolation is performed using GMT’s surface routine (Wessel and Smith 1995) with a tension factor of 0.

### Open boundary conditions

At the open sea boundaries, boundary conditions are defined in the form of water levels. These are composed as the sum of the three main contributors to the total water levels: the astronomical tide, surge, and baroclinic water level variations. To obtain a model that provides water levels with respect to the European Gravimetric Geoid 2015 model (EGG2015) (Denker 2013, 2015) in the mean-tide system, a constant has been added to the prescribed water levels. This constant has been computed from the differences between an observation- and model-derived mean dynamic topography surface (Slobbe et al. 2013a). Note that as we are interested in the differences between the MWL at the mainland and Wadden island tide gauges, the vertical reference of the model-derived water levels is not relevant.

#### The astronomical tide

The astronomical tidal water level ($\zeta _{\text{ a }}(\vartheta ,\lambda ,t)$) is derived by a harmonic expansion using 26 constituents (International Hydrographic Organization—Tides, Water Level And Currents Working Group 2016), namely 5 long-term constituents: *Ssa*, *MSf*, *Mm*, *Mf*, and *Mfm*; 11 diurnal constituents: $2Q_1$, $\sigma _1$, $Q_1$, $\rho _1$, $O_1$, $\chi _1$, $\pi _1$, $P_1$, $K_1$, $\varPhi _1$, and $\theta _1$; and 10 semi-diurnal constituents 2*N*2, $\mu _2$, $N_2$, $\nu _2$, $M_2$, $\lambda _2$, $L_2$, $T_2$, $S_2$, and $K_2$

2 $$\begin{aligned} \zeta _{\text{ a }}(\vartheta ,\lambda ,t)= \sum ^{26}_{i=1} f_i H_i \cos (\omega _i t + (V_0 + u)_i - G_i), \end{aligned}$$

where $f_iH_i$, $\omega _i$, and $G_i$ are the amplitude, angular velocity, and phase of harmonic constituent *i*, respectively; and $(V_0 + u)_i$ links the local time basis to the orbital positions of the Sun, Moon and Earth. The amplitudes and phases of the 5 long-term constituents are taken from the FES2012 tidal atlas solution (Carrère et al. 2012). The amplitudes and phases of the other constituents are the same as those being used in the operational version of the DCSMv6, see Zijl et al. (2013) for further details. The 18.6 year nodal tide cycle is approximated by the nodal coefficients $f_i(t)$ and $u_i(t)$.

**Fig. 2:**
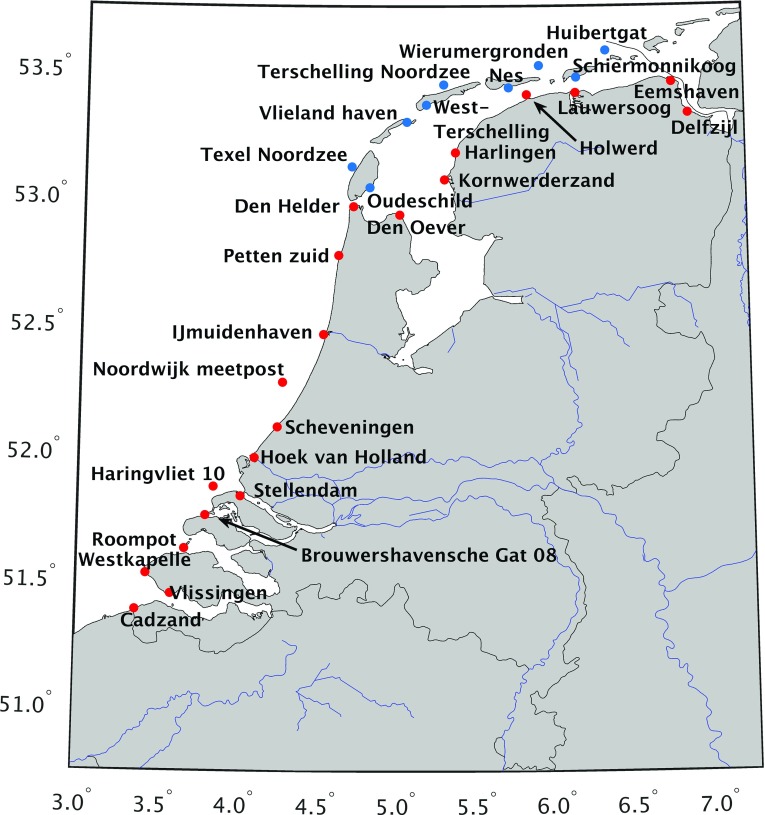
The locations of the 29 Dutch tide gauges directly located along or in the Dutch North Sea or Wadden Sea. The mainland tide gauges are colored red, whereas the Wadden island tide gauges are colored blue

#### Surge

Water level variations induced by the atmospheric wind and pressure forcing are taken from the “Dynamic Atmospheric Correction” product provided by AVISO (CNES/CNRS-Legos/CLS 2016). This product is based on the Mog2D-G high-resolution barotropic model (Carrère and Lyard 2003) for frequencies less than 20 days and the inverted barometer correction otherwise.

#### The baroclinic water levels

The baroclinic water levels are computed from the 25-hourly MWLs that are part of the output of the Atlantic—European North West Shelf—Ocean Physics Reanalysis (kindly provided by John Siddorn) by removing the 25-hourly mean astronomical tidal and surge water levels.

## Tide gauge selection

In this section, we present the selection of the mainland and Wadden islands/ offshore tide gauges between which we establish model-based hydrodynamic leveling connections. This preparatory step is required as for some tide gauges (1) the observation record includes large gaps, and/or (2) the model lacks the physics or resolution to resolve the local MWL. The selection is based on assessments of the total duration of the water level observation records (Sect. 3.1) and the model’s ability to obtain the MWL over the period January 1993–January 2012 at the tide gauge locations (Sect. 3.2). Starting point are all 29 tide gauges directly located along or in the Dutch North Sea, the Wadden Sea, or the Dutch estuaries (see Fig. 2) that (1) are inside the ZUNOv4 model domain, (2) include measurements in our simulation period January 1993–January 2012, and (3) are connected to the NAP height system. For the mainland tide gauges, the latter is a prerequisite to apply hydrodynamic leveling. For the Wadden Island/ offshore tide gauges, a connection to the NAP height system allows to validate the model-derived MWL (differences) assuming vertical land motion can be neglected.

### Assessment of the total duration of the water level observation records

The observation records of all 29 tide gauges include measurements over our simulation period. Ideally, they cover the full simulation period. If not, we can expect differences between the observation- and model-derived MWLs associated with the temporal variations of the mean sea level within the simulation period. Hence, tide gauges whose records include large data gaps over our simulation period should be excluded.

**Fig. 3:**
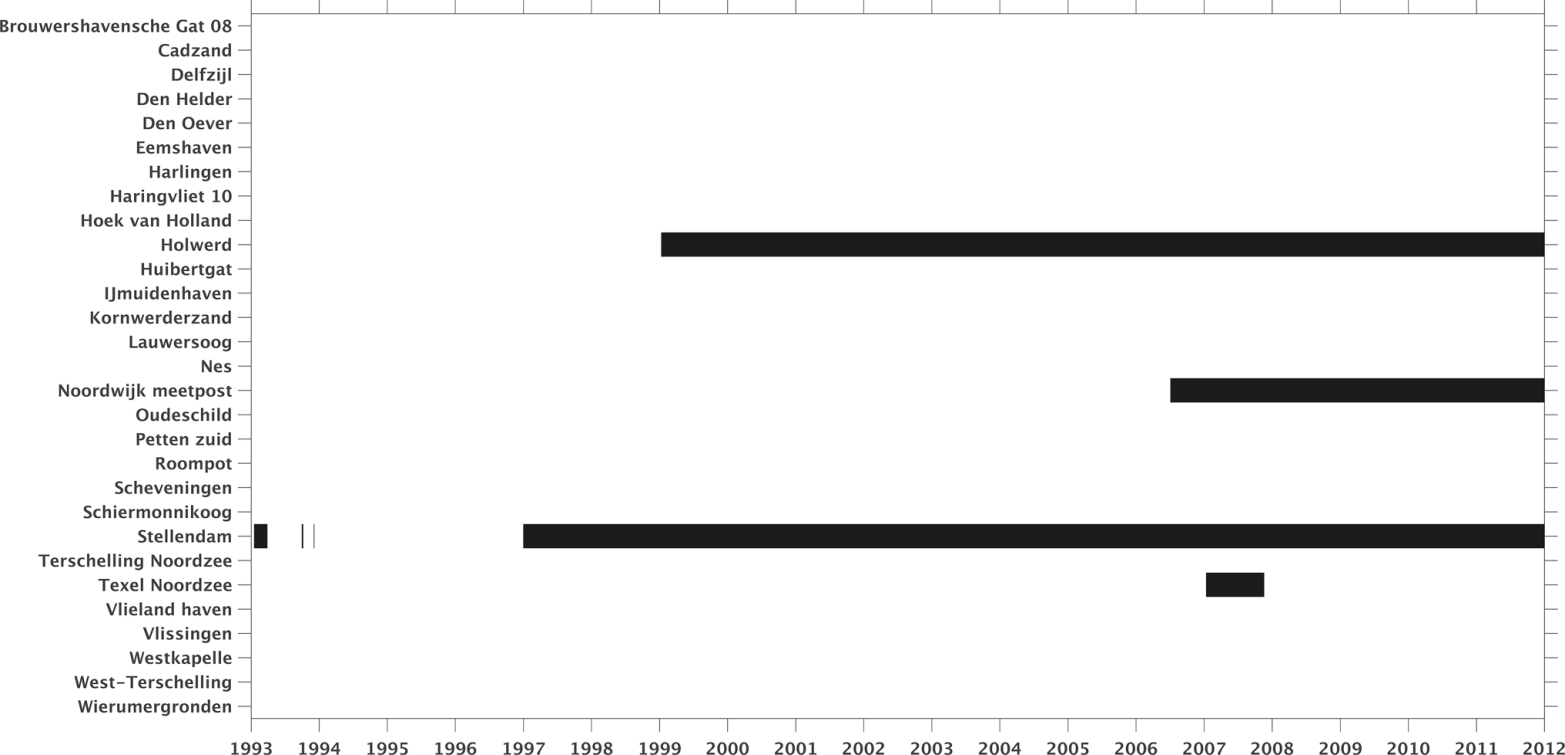
For each tide gauge, the data gaps (black intervals) are shown over the simulation period January 1993–January 2012

Figure 3 shows for each tide gauge the data gaps over the period January 1993–January 2012. In four cases, data gaps are present; Holwerd, Noordwijk meetpost, Stellendam, and Texel Noordzee. Only the latter tide gauge is located at a Wadden island. Since the total duration over which we lack observations for Texel Noordzee is only 1 year, we have included it in the analysis. The other tide gauges are removed from the initial selection.

### Assessment of the model’s ability to obtain the MWL over the period January 1993–January 2012 at the tide gauge locations

The second selection criterion is that the hydrodynamic model should provide a proper representation of the local MWL. At some locations, this cannot be expected because the DCSMv6-ZUNOv4 (1) does not include all relevant physics, and/or (2) lacks the required resolution to resolve the local processes. In this subsection, we identify the tide gauges to which this applies by a comparison of the model- and observation-derived MWLs over the period January 1993–January 2012 (referred to as the “19-year MWL”) at the tide gauge locations. For reference, we start with a brief discussion of the model-derived 19-year MWL signal itself.

**Fig. 4:**
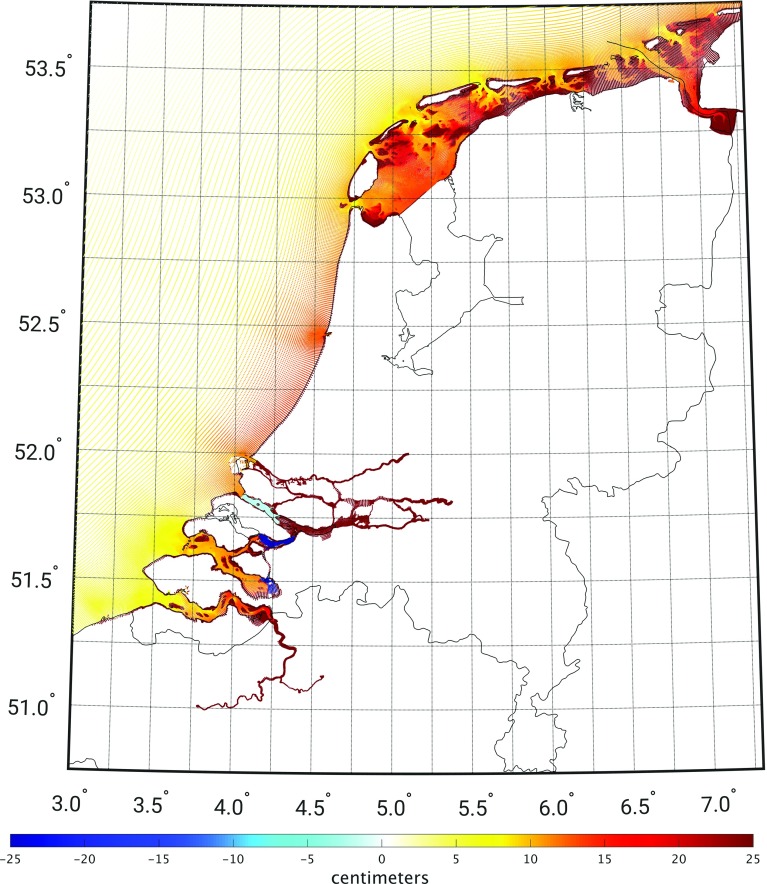
The model-derived MWL relative to the EGG2015 along the Dutch coast and in the Wadden Sea computed using the DCSMv6-ZUNOv4 model over the period January 1993 till January 2012

Figure 4 shows the model-derived 19-year MWL, computed over the period January 1993–January 2012. The 19-year MWL shows a north–west to south–east tilt going from 0 cm in the north–west corner to values up to 14 cm near the Dutch coast around Scheveningen (cf. Fig. 2). Toward the south, the values decrease to about 7 cm. Toward the north, the 19-year MWL is about 10 cm along the North Sea side of the Wadden islands Texel, Vlieland, and Terschelling. Further to the east it again increases. The model-derived 19-year MWL signal on the North Sea is consistent with oceanographic expectations. The overall gradient can be explained by both prevailing winds and baroclinic signals (Slobbe et al. 2013a). The feature between $52^\circ$ and $53^\circ$ is mainly the contribution of tides to the MWL (cf. Prandle 1978; Slobbe et al. 2013b). In the Wadden Sea, the signal should be interpreted with care. During low tides, large parts of the Wadden Sea fall dry (these are the so-called tidal flats). If this is the case, the corresponding cells become inactive and no water levels are computed. The 19-year MWL is computed as the mean over the times spans the cell is wet.

**Fig. 5:**
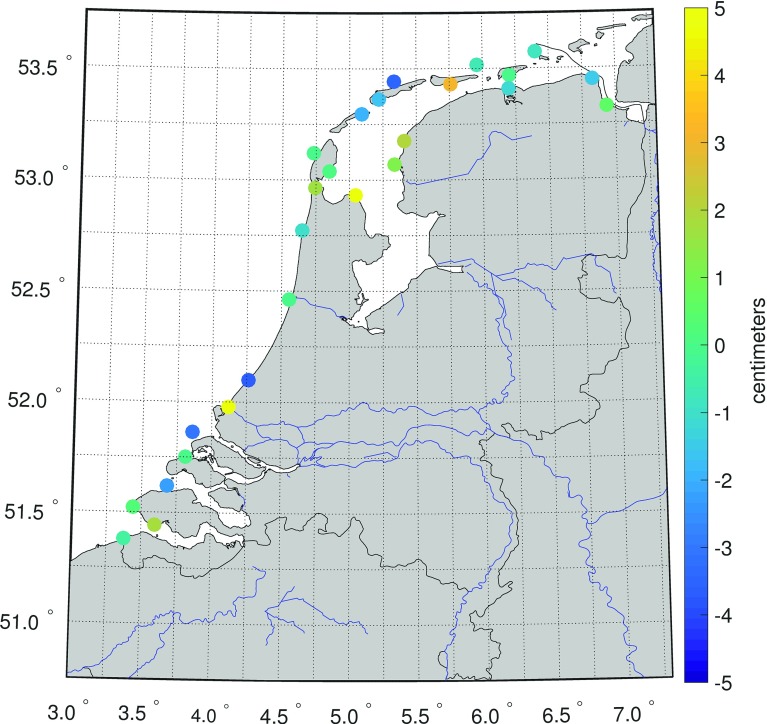
The differences between the observation-derived and model-derived MWLs at all Dutch tide gauges. Both are computed over the period January 1993 till January 2012. Note that the mean difference is removed; as we are interested in the differences between the MWL at the tide gauges rather than in the MWL itself this bias cancels out

To assess the quality of the model-derived 19-year MWL values, we compare them at the tide gauge locations to the 19-year MWL computed from the tide gauge records. A spatial rendition of the differences is shown in Fig. 5. This figure shows an anomalous behavior at two tide gauges; Hoek van Holland and Den Oever with differences of 6.8 and 4.9 cm, respectively. Regarding Hoek van Holland, we can explain the misfit by the fact that our model does not resolve the processes associated with freshwater discharge. This tide gauge is located at the mouth of the Rotterdam Waterway. Here, every ebb tide lenses of freshwater are ejected by the Rhine and Meuse rivers. They spread forming ever larger lenses that merge and interact with the lenses emitted on the previous tidal cycles forming the “Rhine Region of Freshwater Influence” (de Boer et al. 2006, 2008). It is dynamic, with significant tidal currents modified by baroclinic effects, due to the highly variable temperature and salinity. The 2D barotropic model used in our study neither captures this variability nor resolves the strongly sheared counter-rotating tidal currents (de Boer et al. 2009).

**Fig. 6:**
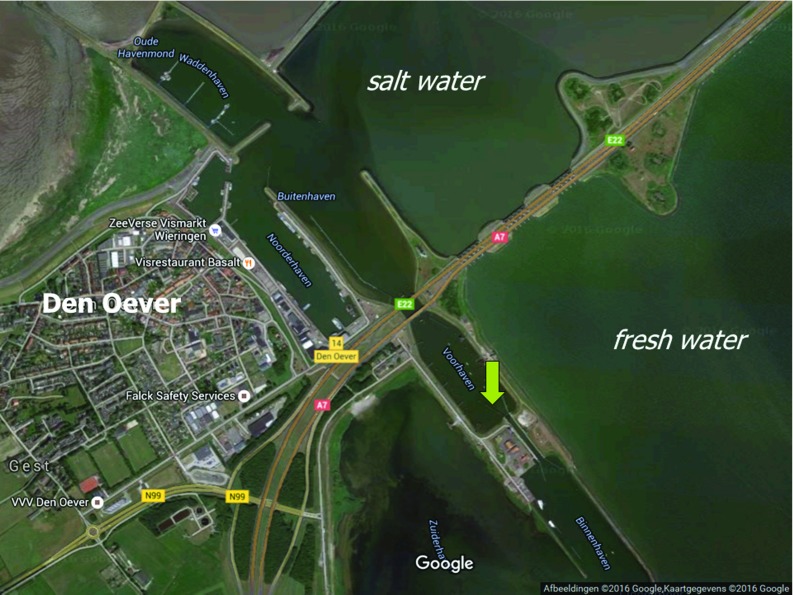
Location of tide gauge Den Oever (green arrow). Background image taken from http://maps.google.nl

In case of the tide gauge Den Oever, we attribute the misfit to local conditions not captured by the model. The tide gauge is located inside the Stevin sluice complex that connects the Wadden Sea to Lake IJssel (see Fig. 6). The model lacks the resolution to resolve the dynamics in and around this sluice complex. Apart from this, the location is exactly at the interface of fresh (Lake IJssel) and salt water (Wadden Sea). As mentioned earlier, the model does not resolve the processes associated with freshwater discharge. Hence, we can expect a poor representation of the MWL also at this location.

## Results and discussion

In this section, we present, discuss, and validate the hydrodynamic leveling data obtained by computing the differences in the model-derived MWL at the mainland and Wadden island tide gauges. The most obvious choice of the averaging period is to use the full simulation period, i.e., January 1993–January 2012 (Sect. 4.1). This time span covers one full nodal cycle (18.6 years). However, during the storm surge period (the autumn and winter months), larger model errors can be expected. In Sect. 4.2, we analyze the differences between the model-based hydrodynamic leveling data obtained using the strategy where we compute the MWL over the full simulation period (referred to as the “19-year MWL”) and the strategy in which only the summer months over the full simulation period are used (referred to as the “19-year summer MWL”). Finally, we assess what impact the length of the time span has on the model’s ability to reconstruct the differences in the MWL.

### Model-based hydrodynamic leveling based on the 19-year MWL

**Fig. 7:**
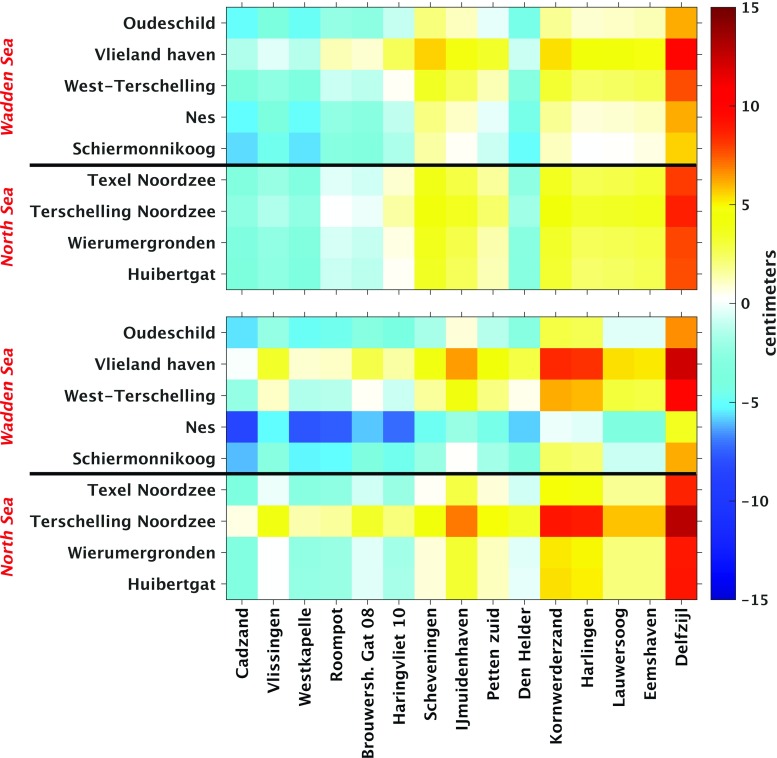
Model-derived (DCSMv6-ZUNOv4) differences in the MWL (top panel) and observation-derived differences in the MWL (bottom panel) among the Dutch mainland tide gauges and tide gauges at or nearby the Dutch Wadden islands. The MWLs are computed over the period January 1993–January 2012. The mainland tide gauges are sorted by their location when traveling along the coast line from the south to the north. The Wadden island tide gauges are sorted similarly, but grouped in those located along the Wadden Sea side and those located along the North Sea side of the Wadden islands

The model-derived 19-year MWL differences are shown in the top panel of Fig. 7. In row direction, i.e., from the south to the north, the differences show a rather smooth trend from about $-\,4$ to $+\,4$ cm. Only the 19-year MWL differences computed from tide gauge Den Helder interrupt this trend. This anomalous behavior is explained by the fact that tide gauge Den Helder is located in the Marsdiep, a deep tidal-race between Den Helder and the Wadden island Texel that connects the North Sea and the Wadden Sea. Looking columnwise, we do not observe much variations for the Wadden island tide gauges located at the North Sea side; i.e., the 19-year MWL does hardly change when going from Texel to Huibertgat. The only exception is Terschelling Noordzee. For the Wadden island tide gauges located at the Wadden Sea side, more variation is observed. From an hydrodynamics point of view, this is expected; the Wadden Sea has a complex bathymetry (see Fig. 1c), which should be reflected by stronger local variations in the MWL.

**Fig. 8:**
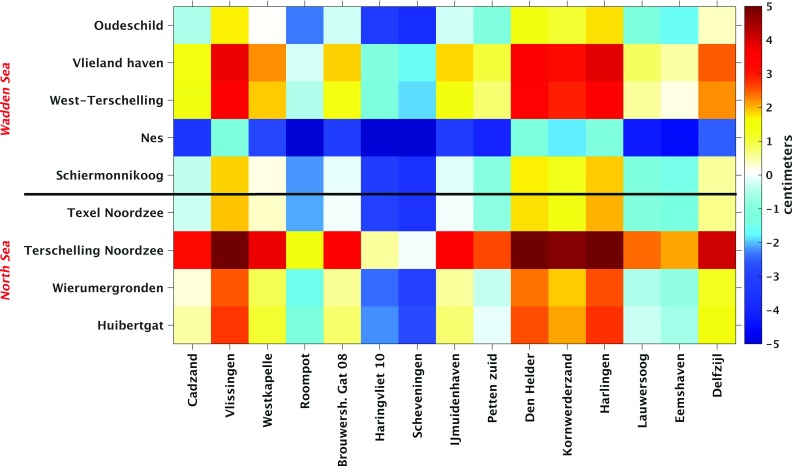
Difference between the observation-derived differences in 19-year MWL (bottom panel Fig. 7) and the model-derived differences in 19-year MWL (top panel Fig. 7) among Dutch mainland tide gauges and tide gauges at or nearby the Dutch Wadden islands

To validate the model-derived hydrodynamic leveling data, we compared them to their counterpart derived from the observed tide gauge records (bottom panel of Fig. 7). In general, the two figures show a good agreement; the overall trend is the same and has the same order of magnitude. The observation-derived 19-year MWL differences show, however, larger variations compared to the model-derived ones. To facilitate the comparison, we subtracted the model-derived differences from the observation-derived ones. The resulting discrepancies are shown in Fig. 8. The largest discrepancies show up for the tide gauges Nes and Terschelling Noordzee. In both cases, the mismatch seems to be rather constant; negative for Nes while positive for Terschelling Noordzee. This suggests the problem is with the Wadden island tide gauges. For tide gauge Nes, this again might be explained by the fact that the model is not capable to resolve the local MWL. At the same time, however, we cannot exclude the possibility of a local vertical motion of these tide gauges.

**Fig. 9:**
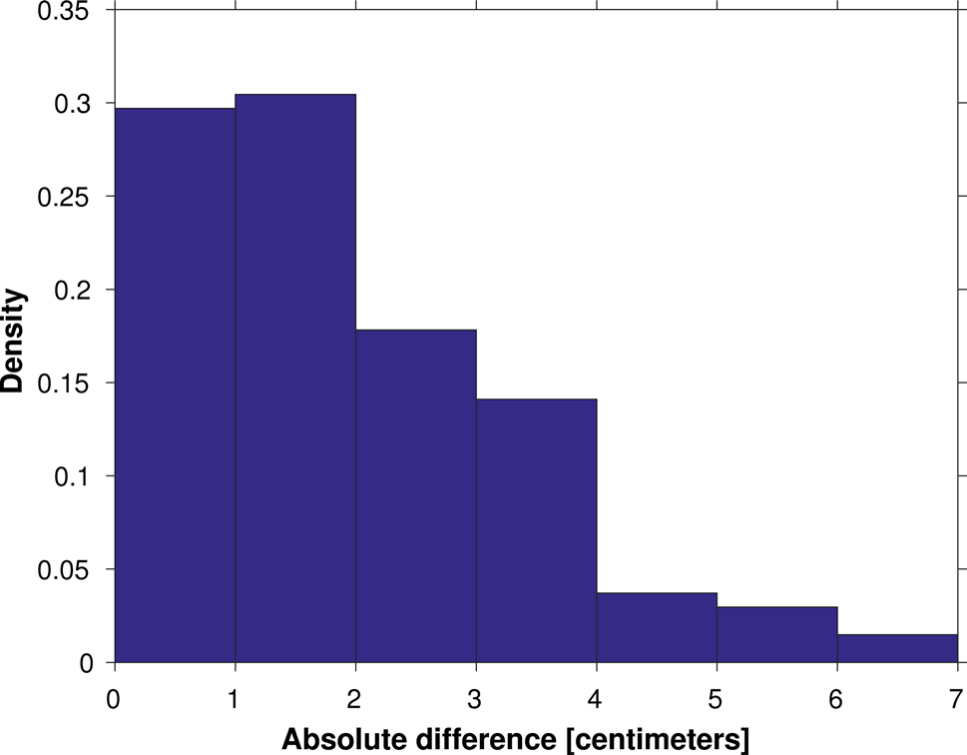
Normalized histogram of the absolute differences between the observation- and model-derived differences in 19-year MWL among Dutch mainland tide gauges and tide gauges at or nearby the Dutch Wadden islands (shown in Fig. 8)

Based on the normalized histogram of the absolute differences between the observation- and model-derived 19-year MWL differences shown in Fig. 9, we conclude that about 30% of all absolute differences are $\le 1$ cm and about 60% are $\le 2$ cm. These numbers correspond to 40 and 81 of the 135 possible leveling connections, respectively. Except for tide gauge Nes, for each Wadden island tide gauge there are several connections for which the absolute difference is $\le 1$ cm.

**Fig. 10:**
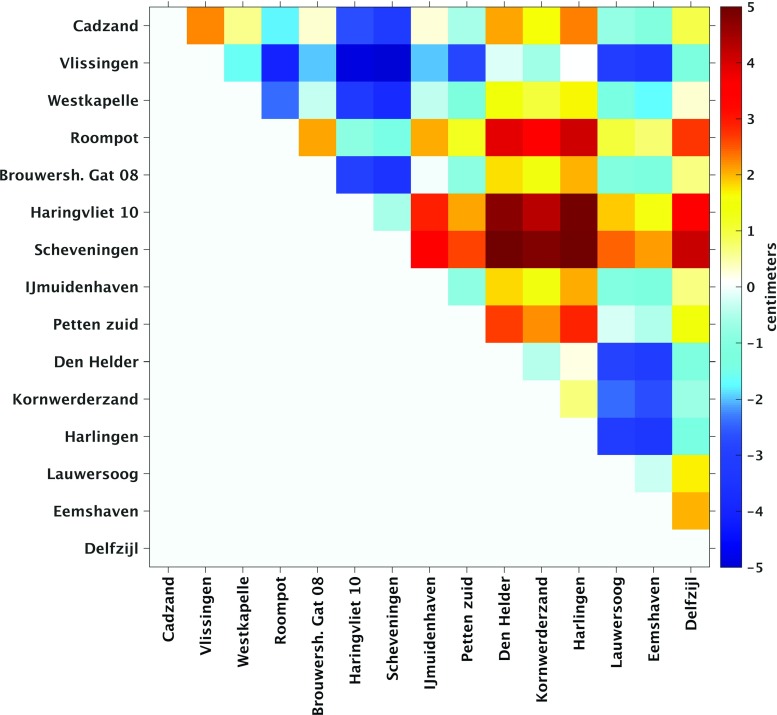
Difference between the observation-derived differences in 19-year MWL and the model-derived differences in 19-year MWL among Dutch mainland tide gauges

**Fig. 11:**
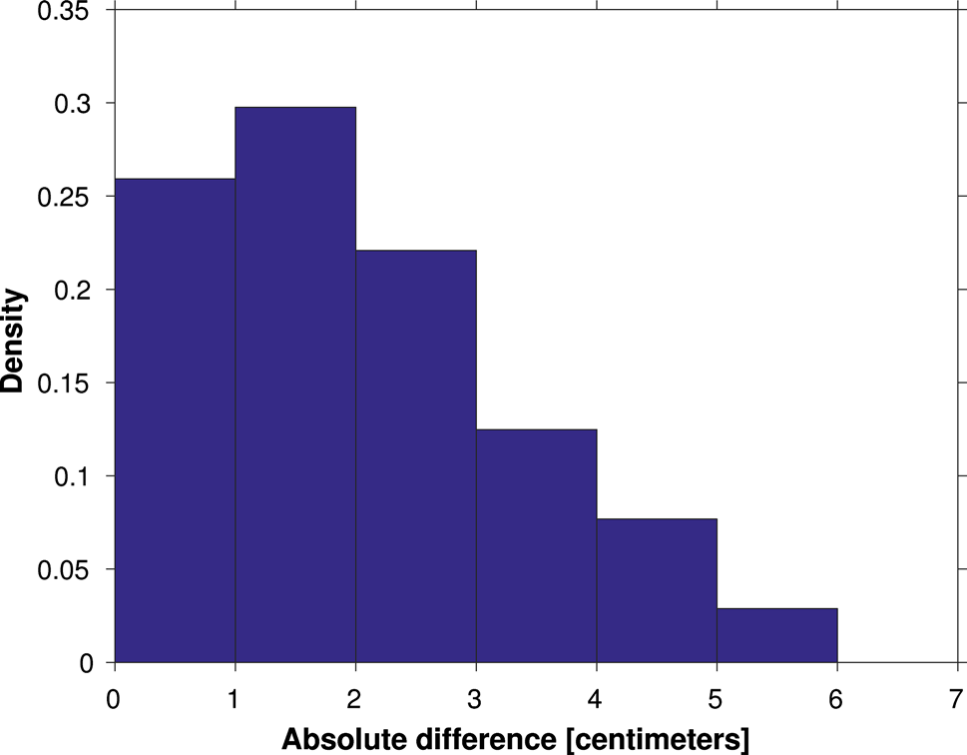
Normalized histogram of the absolute differences between the observation- and model-derived differences in 19-year MWL among Dutch mainland tide gauges and tide gauges at or nearby the Dutch Wadden islands (shown in Fig. 10)

We cannot exclude the possibility of (local) vertical motions of the Wadden island tide gauges. (Their benchmarks have not been re-leveled for at least 15 years.) If there were motions, they would contaminate the observation-derived 19-year MWL differences and in turn reduce the number of connections for which the absolute difference is $\le 1$ cm. The mainland tide gauges are not affected by this problem. Hence, we repeated the above-described analysis using the mainland tide gauges only. Figure 10 shows the differences between the observation- and model-derived 19-year MWL differences among the Dutch mainland tide gauges, whereas Fig. 11 shows the normalized histogram of the absolute values of these differences. We find that the percentage of absolute differences $\le 1$ cm is about 4% lower, while the percentage of absolute differences between 1 and 2 cm is similar. Therefore, we conclude that in general discrepancies between the observation- and model-derived 19-year MWL differences are not dominated by signals associated with local vertical motions of the Wadden island tide gauges.

### Model-based hydrodynamic leveling based on the 19-year summer MWL

**Fig. 12:**
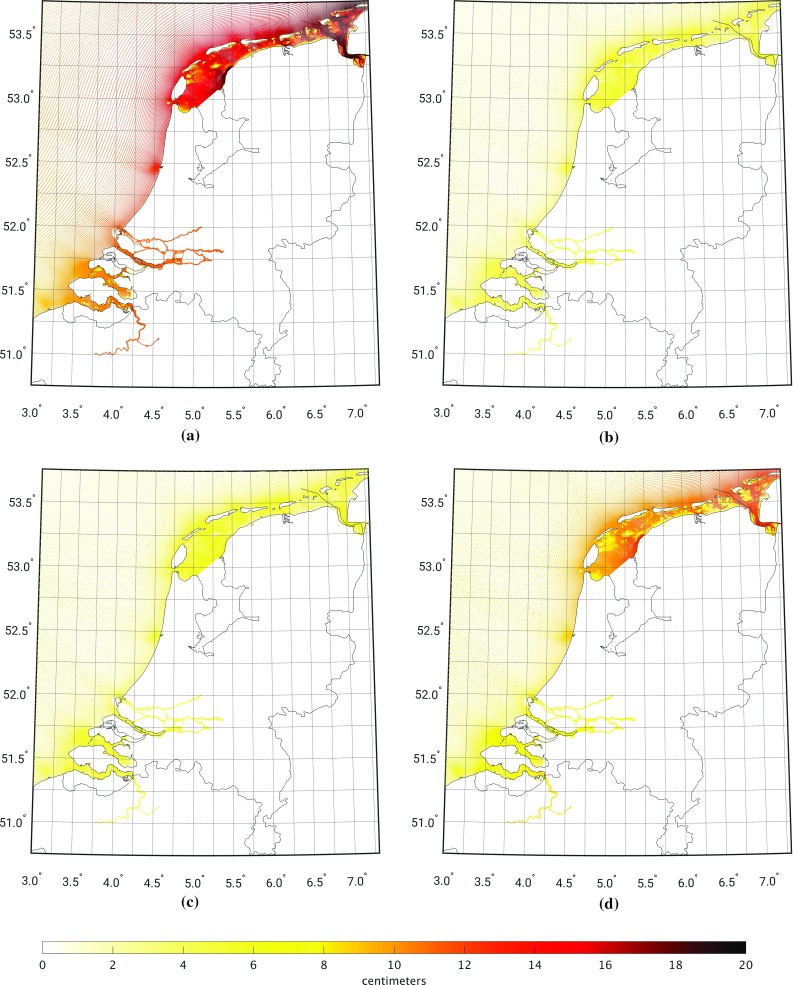
Standard deviation of the model-derived monthly MWLs compared to the seasonal MWL for the winter (**a**), spring (**b**), summer (Standard deviation ), and fall (**d**). In computing the standard deviation, we defined the winter as the period from January 1–April 1, spring as April 1–July 1, summer as July 1–October 1, and fall as October 1–January 1

**Fig. 13:**
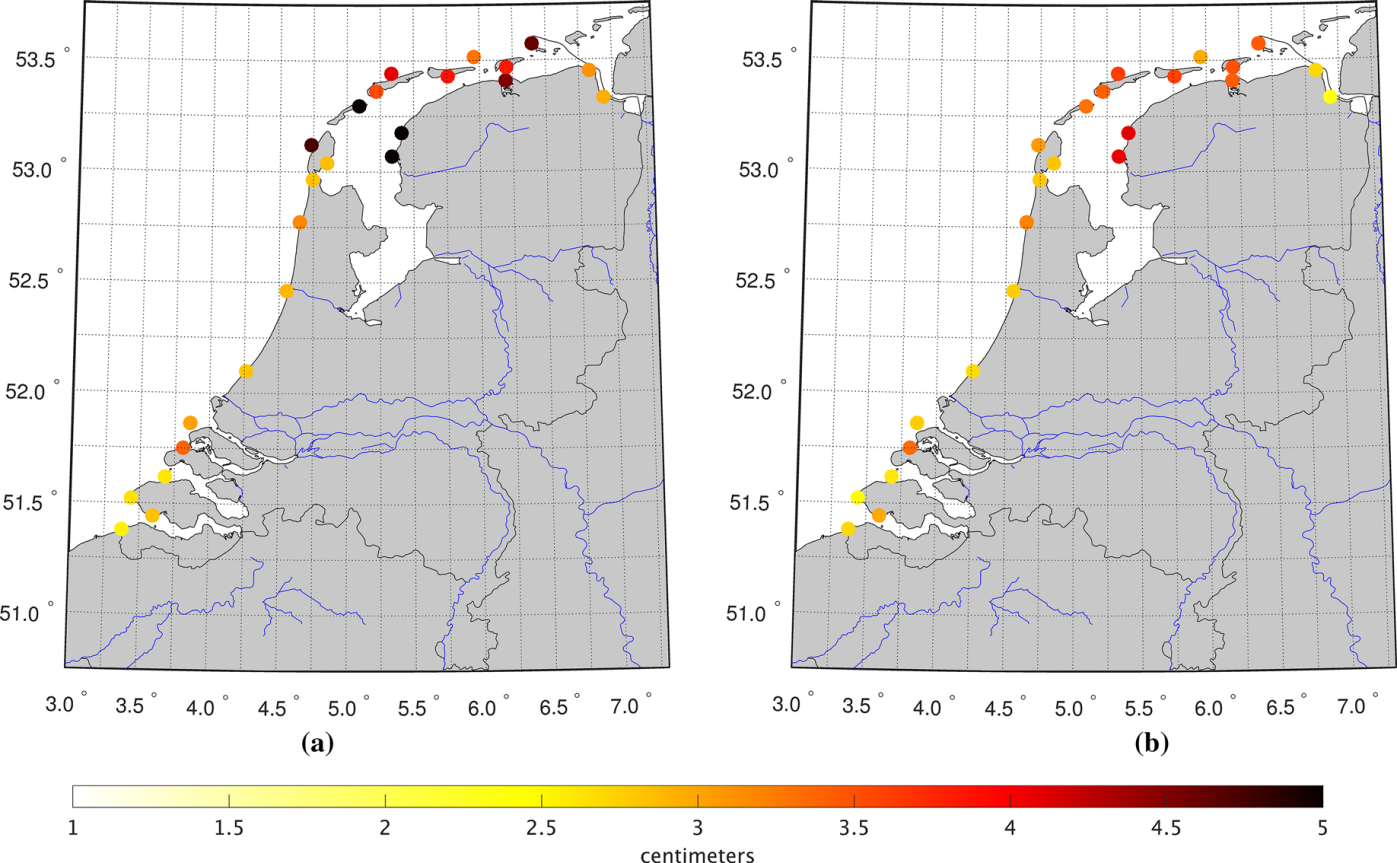
Standard deviation of the differences between the observation- and model-derived monthly MWLs evaluated at the tide gauges for the winter (**a**) and summer (**b**) seasons. In computing the standard deviation, we defined the winter as the period from January 1–April 1, and summer as July 1–October 1

The variability of the sea level is not the same throughout the year. During the autumn and winter seasons, the water level inside the North Sea is often perturbed by storm surges that can cause deviations lasting several days up to meters in the Dutch coastal waters. To illustrate this difference in variability over monthly time scales, we compute for all seasons the standard deviation of the model-derived monthly MWLs per model grid point. The standard deviations are shown in Fig. 12. The differences are significant; during winter the model-derived monthly MWLs in the Wadden Sea have a standard deviation of about 15 cm, whereas in summer the variability is just about 5.5 cm. Since the spatiotemporal resolution of the used wind and mean sea level pressure data (see Sect. 2.2.2) is pretty coarse, we expect the poorest model performance when the meteorological conditions are rough, i.e., during winter. This expectation is confirmed by Fig. 13, which shows for the winter and summer seasons the standard deviation of the differences between the observation- and model-derived monthly MWLs evaluated at the tide gauges. In particular in the north of the Netherlands the standard deviations for the winter season are significantly larger.

**Fig. 14:**
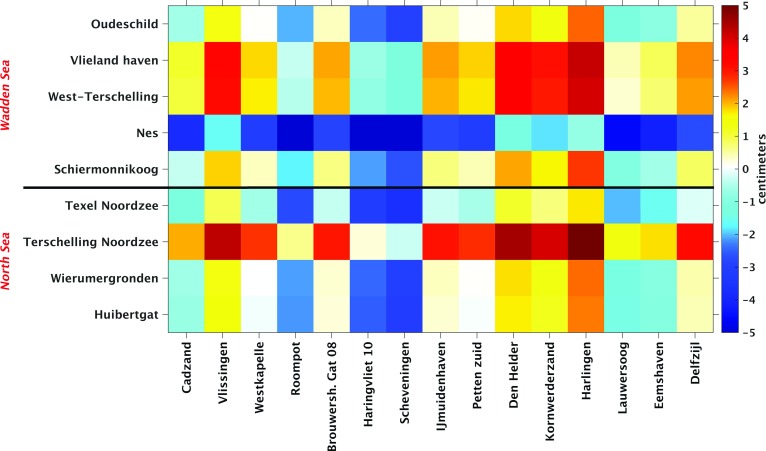
Difference between the observation- and model-derived 19-year summer MWL differences among the Dutch mainland tide gauges and tide gauges at or nearby the Dutch Wadden islands

**Fig. 15:**
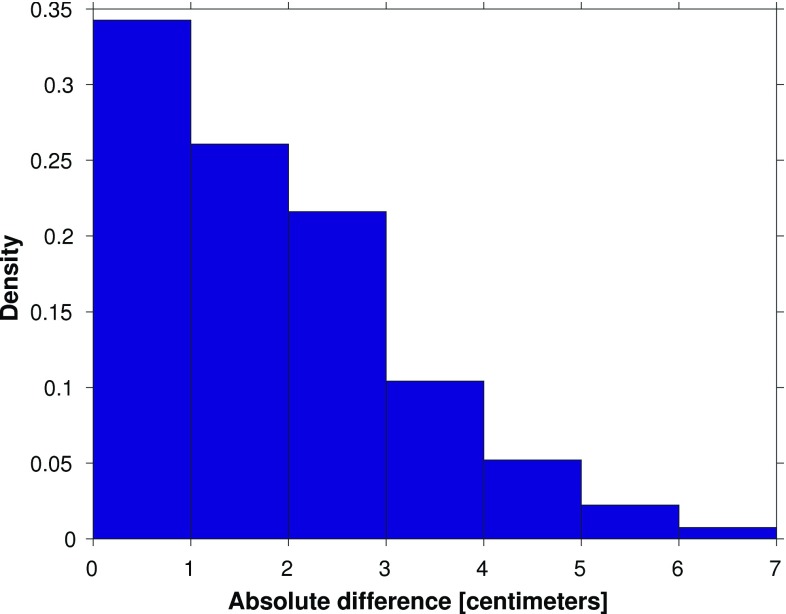
Normalized histogram of the absolute differences between the observation- and model-derived differences in 19-year summer MWL among Dutch mainland tide gauges and tide gauges at or nearby the Dutch Wadden islands (shown in Fig. 14)

The results suggest that the performance of model-based hydrodynamic leveling may increase when using only the summer months over the 19-year simulation period to compute the MWL (i.e., the 19-year summer MWL) instead of using all months. To prove whether this is indeed the case, we repeat the analysis presented in the previous section. In Fig. 14, we show the differences between the observation- and model-derived 19-year summer MWL differences among the tide gauges (cf. Fig. 8) and in Fig. 15 the normalized histogram of the absolute differences (cf. Fig.9). By comparing Figs. 8 and 14, we conclude that indeed the differences between the observation- and model-derived 19-year summer MWL differences are lower compared to those obtained with the 19-year MWL. In the histogram (Fig. 15), we can clearly observe that the peak has shifted toward the left; about 34% (46 of the 135 possible leveling connections) of all absolute differences are $\le 1$ cm compared to about 30% (40 out of 135) when using the 19-year MWL. Using this setup, for *all* Wadden island tide gauges several connections can be found for which the absolute difference is $\le 1$ cm.

The impact of averaging over the months of a particular season only, becomes even more pronounced when we compare the results obtained when using the summer months to those obtained using the winter months (not shown here). In the latter case, only about 26% (35 of the 135 possible leveling connections) of all absolute differences are $\le 1$ cm. In the remainder of this manuscript, we adopt the strategy to use the summer months only in computing the MWL.

### What is the impact of the simulation period length?

**Fig. 16:**
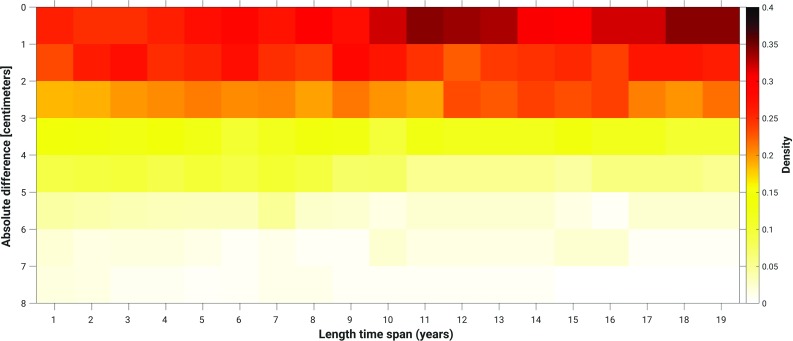
Normalized histogram of the absolute differences between the observation- and model-derived summer MWL differences among the Dutch mainland tide gauges and tide gauges at or nearby the Dutch Wadden islands as a function of the length of the time span

**Fig. 17:**
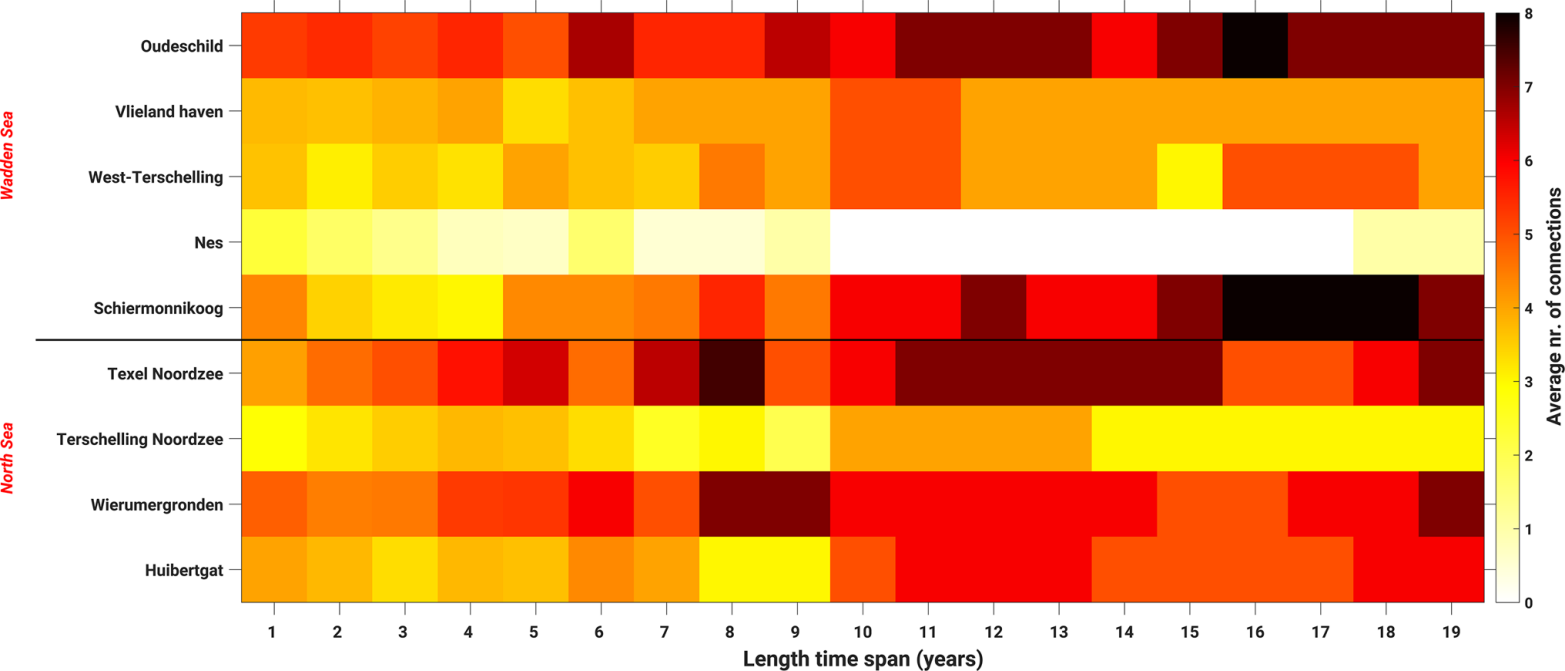
Average number of connections to all Wadden island tide gauges for which the differences between the observation- and model-derived summer MWL differences are $\le 1$ cm. Note that the maximum number of possible connections for all Wadden island tide gauges equals the total number of mainland tide gauges, i.e., 15

So far, the MWL is computed over one full nodal cycle (Sect. 4.1) or all summer months over one full nodal cycle (Sect. 4.2). Both choices guarantee that the signal associated with the differences between the nodal cycle at the two tide gauge locations between which the differences in MWL are computed, averages out. As mentioned before, to conduct the leveling modeled water levels are only needed at one epoch. The motivation to use the *average* water level over a long time span is to suppress “high-frequency” noise. At the same time, however, we implicitly assume that “local” conditions remain unchanged. For example, we assume a static bathymetry over the entire simulation period. In reality, this is not the case. In particular, the Wadden Sea exhibits an ever-changing morphology (topography/bathymetry) of the islands, tidal channels, inter-tidal shoals, and tidal flats (Wang et al. 2012). Therefore, one would expect that depending on the connection, shorter periods than the full nodal cycle may provide even better results. In this section, we assess the impact of using a shorter time span. In doing so, we compute the summer MWLs over time spans varying from 1 to 19 years in steps of 1 year over the period January 1993–January 2012. Following Frederikse et al. (2016), we accounted for the differences in the MWL among the tide gauges associated with the differences in the nodal cycle by assuming that the amplitude follows the self-consistent equilibrium law and no phase shift occurs (Woodworth 2012). For each time span, we compute the normalized histogram of the absolute differences between the observation- and model-derived summer MWL differences among the Dutch mainland tide gauges and tide gauges at or nearby the Dutch Wadden islands. In case we can choose more than one sub-period of the original simulation period January 1993–January 2012, we compute the average normalized histogram. The (averaged) normalized histograms are shown in Fig. 16. In Fig. 17, we show the (average) number of connections to all Wadden island tide gauges for which the absolute differences between the observation- and model-derived summer MWL differences are $\le 1$ cm as a function of the length of the time span. The histograms show that the differences between the observation- and model-derived summer MWL differences are lowest when the period length is either around 11 years or between 18 and 19 years. Overall, however, the differences are not large for time spans larger than 10 years. From Fig. 17, we conclude that for all tide gauges the largest number of connections with differences $\le 1$ cm is or can already be obtained when computing the summer MWL over a shorter time span than the full 19 years. At the same time, the “optimum” simulation period length is different for most tide gauges. The question how these results can be explained from an oceanographic point of view is out of the scope of this study. We conclude that the period length over which the (summer) MWLs are computed is important to the performance of model-based hydrodynamic leveling. In general, a period length longer than 10 years is the better choice.

## Summary and conclusions

In this study, we presented an efficient and flexible alternative method to connect islands and offshore tide gauges with the height system on land. The method exploits a regional, high-resolution hydrodynamic model providing total water levels to compute the differences in MWL between tide gauges at the mainland and at the islands or offshore platforms. Adding them to the MWL relative to the national height system at the mainland’s tide gauges realizes a connection of the island and offshore platforms with the height system on the mainland. The method is applied to connect the Dutch Wadden islands to the national height system. In this study, we used a model that consists of two domains that are coupled by means of domain decomposition. In the outer domain, the Dutch Continental Shelf Model version 6 (DCSMv6) (Zijl et al. 2013) is used and in the inner domain, the Zuidelijk Noordzee model version 4 (ZUNOv4). This barotropic (2D) model is primarily developed to forecast storm surges. We included the baroclinic forcing by adding the depth-averaged horizontal baroclinic pressure gradients as a diagnostic variable. The main research question addressed in this study is whether hydrodynamic leveling based on such a model allows to transfer NAP from the Dutch mainland to the Wadden islands and nearby offshore platforms with centimeter accuracy.

To answer this question, several choices of the period over which the MWLs are computed are tested and validated. In the most straightforward implementation of model-based hydrodynamic leveling, the MWL differences are computed by averaging *all* water levels over the full simulation period (in this study, January 1993–January 2012). Using this strategy, for about 30% of all connections (40 out of 135) the absolute differences between observation- and model-derived MWL differences are $\le 1$ cm. For two out of nine Wadden island tide gauges, the discrepancies are quite systematic. Except for tide gauge Nes, for each Wadden island tide gauge there are several connections for which the absolute difference between the observation- and model-derived MWL is $\le 1$ cm.

Next, we computed the MWL over the summer months of the 19-year simulation period only. Based on this strategy, the percentage of connections for which the absolute differences between the observation- and model-derived MWL differences are $\le 1$ cm increases to about 34% (46 out of 135). In this case, for all Wadden island tide gauge there are several connections for which the absolute difference is $\le 1$ cm.

Finally, we investigated the impact of the period length over which the MWL is computed. The choice of this period length turns out to be important to the performance of model-based hydrodynamic leveling. We found that for all tide gauges the largest number of connections with differences $\le 1$ cm is or can already be obtained when computing the summer MWL over a shorter time span than the full 19 years. At the same time, the “optimum” simulation period length is different for most tide gauges. A period length longer than 10 years seems to be the better choice.

Based on these results, we can answer the main research question positively. Whether or not the presented approach allows to achieve the same performance in other ocean areas remains to be investigated. Provided all input data are available, there are no intrinsic limitations that do not allow to apply the technique elsewhere. In our opinion, the results show that model-based hydrodynamic leveling has a great potential. In a future work, we will develop and apply a hydrodynamic model that resolves *all* relevant 3D physical processes (including freshwater discharge). We believe that this will further improve the quality of the data and allows to include even more tide gauges. In addition, we will apply the technique to cross the North Sea in order to strengthen the United European Leveling Network that is used to realize the European Vertical Reference System. The strengthening will improve the accuracy of a realization of this system, which is specified by INSPIRE as the height reference system for pan-European geo-referencing.
